# Pursuing Equity: The Struggle Continues

**DOI:** 10.1016/j.atssr.2023.11.001

**Published:** 2023-11-21

**Authors:** Dane C. Paneitz, Jordan P. Bloom

**Affiliations:** Department of Surgery, Johns Hopkins University School of Medicine, Baltimore, Maryland; Division of Cardiac Surgery, Massachusetts General Hospital, 55 Fruit St, Cox 630, Boston, MA 02114

Reply To the Editor:

We thank Arrington and colleagues for their interest in and response to our survey that examined early career experiences of cardiothoracic surgery graduates between 2012 and 2020 stratified by the type of training model completed.^1^^,^^2^ Arrington and colleagues note the underrepresentation of female and Black or African American cardiothoracic surgery graduates in our study and call for increased intentional efforts to recruit and to retain women and underrepresented minorities into the field of cardiothoracic surgery, which we also support. Although there is still much to be done, it is encouraging that a study published by 1 of the coauthors in 2022 found that both the proportions of trainees who are female and trainees of underrepresented races/ethnicities increased between 2011 and 2019.^3^ This is likely in part due to the work and growth of the Women in Thoracic Surgery, Association of Black Cardiovascular and Thoracic Surgeons, and efforts of The Society of Thoracic Surgeons and The American Association for Thoracic Surgery that should be continued and strengthened.
